# High Correlation of Static First-Minute-Frame (FMF) PET Imaging after ^18^F-Labeled Amyloid Tracer Injection with [^18^F]FDG PET Imaging

**DOI:** 10.3390/s21155182

**Published:** 2021-07-30

**Authors:** Alexander P. Seiffert, Adolfo Gómez-Grande, Alberto Villarejo-Galende, Marta González-Sánchez, Héctor Bueno, Enrique J. Gómez, Patricia Sánchez-González

**Affiliations:** 1 Biomedical Engineering and Telemedicine Centre, ETSI Telecomunicación, Center for Biomedical Technology, Universidad Politécnica de Madrid, 28040 Madrid, Spain; enriquejavier.gomez@upm.es; 2 Department of Nuclear Medicine, Hospital Universitario 12 de Octubre, 28041 Madrid, Spain; adolfo.gomez@salud.madrid.org; 3 Facultad de Medicina, Universidad Complutense de Madrid, 28040 Madrid, Spain; alberto.villarejo@salud.madrid.org (A.V.-G.); hector.bueno@cnic.es (H.B.); 4 Department of Neurology, Hospital Universitario 12 de Octubre, 28041 Madrid, Spain; mgonzalezsanchez2@salud.madrid.org; 5 Group of Neurodegenerative Diseases, Hospital 12 de Octubre Research Institute (imas12), 28041 Madrid, Spain; 6 Biomedical Research Networking Center in Neurodegenerative Diseases (CIBERNED), 28029 Madrid, Spain; 7 Department of Cardiology and Instituto de Investigación Sanitaria (imas12), Hospital Universitario 12 de Octubre, 28041 Madrid, Spain; 8 Centro Nacional de Investigaciones Cardiovasculares (CNIC), 28029 Madrid, Spain; 9 Centro de Investigación Biomédica en Red de Enfermedades Cardiovasculares (CIBERCV), 28029 Madrid, Spain; 10 Centro de Investigación Biomédica en Red de Bioingeniería, Biomateriales y Nanomedicina (CIBER-BBN), 28029 Madrid, Spain

**Keywords:** FMF, amyloid PET, early-phase, florbetapir, flutemetamol, florbetaben, Alzheimer’s disease, mild cognitive impairment, neurodegeneration biomarker

## Abstract

Dynamic early-phase PET images acquired with radiotracers binding to fibrillar amyloid-beta (Aβ) have shown to correlate with [^18^F]fluorodeoxyglucose (FDG) PET images and provide perfusion-like information. Perfusion information of static PET scans acquired during the first minute after radiotracer injection (FMF, first-minute-frame) is compared to [^18^F]FDG PET images. FMFs of 60 patients acquired with [^18^F]florbetapir (FBP), [^18^F]flutemetamol (FMM), and [^18^F]florbetaben (FBB) are compared to [^18^F]FDG PET images. Regional standardized uptake value ratios (SUVR) are directly compared and intrapatient Pearson’s correlation coefficients are calculated to evaluate the correlation of FMFs to their corresponding [^18^F]FDG PET images. Additionally, regional interpatient correlations are calculated. The intensity profiles of mean SUVRs among the study cohort (*r* = 0.98, *p* < 0.001) and intrapatient analyses show strong correlations between FMFs and [^18^F]FDG PET images (*r* = 0.93 ± 0.05). Regional VOI-based analyses also result in high correlation coefficients. The FMF shows similar information to the cerebral metabolic patterns obtained by [^18^F]FDG PET imaging. Therefore, it could be an alternative to the dynamic imaging of early phase amyloid PET and be used as an additional neurodegeneration biomarker in amyloid PET studies in routine clinical practice while being acquired at the same time as amyloid PET images.

## 1. Introduction

Three main biomarkers have been established for the diagnosis of Alzheimer’s disease (AD): (1) biomarkers of amyloid-beta (Aβ) plaques; (2) biomarkers of fibrillar tau; and (3) biomarkers of neurodegeneration [1]. Abnormal extracellular Aβ deposition leads to neuronal death and, consequently, impaired cognitive functions [2]. PET imaging allows for the in vivo evaluation of the deposition of Aβ plaques. Validated Aβ-binding tracers are [^11^C]Pittsburgh compound-B (PiB) [3], [^18^F]florbetapir (FBP) [4], [^18^F]florbetaben (FBB) [5], and [^18^F]flutemetamol (FMM) [6]. Conversely, neuronal impairment and decreased neuronal activity results in reduced brain metabolism [7]. [^18^F]fluorodeoxyglucose (FDG) is currently widely used for the imaging of brain metabolism and the diagnosis of neurodegenerative diseases. Brain [^18^F]FDG PET is regarded as a good biomarker of neurodegeneration in AD [8].

Early-phase brain amyloid PET images haven been shown to correlate positively with [^18^F]FDG and [^15^O]H_2_O brain PET images and offer perfusion information. The studies investigating early-phase brain amyloid PET images use mainly [^11^C]PiB [9,10,11,12,13,14,15,16,17,18,19,20,21], [^18^F]FBP [22,23,24,25,26,27], and [^18^F]FBB [19,28,29,30,31]. In the case of [^18^F]FMM, the dual-time protocol has been studied [32] and the perfusion information has previously been evaluated [33] but early-phase [^18^F]FMM has not been compared to [^18^F]FDG. Dynamic brain PET scans are usually acquired during the 10 min after intravenous injection of the radiotracer. Perfusion images can be generated as the sum or average of the individual dynamic images for radiotracer-specific time frames. While dynamic scans may offer higher accuracy, static PET images are still recommended in clinical guidelines for [^18^F]FDG PET brain imaging and considered sufficient for diagnostic purposes [34,35]. In brain amyloid imaging, dynamic scans are not required clinically and static scans may be used for visual and semi-quantitative interpretation [36]. Moreover, the evaluated dynamic early-phase protocols would require at least an additional 5 min of scan time, while an easier-to-acquire static scan of a fixed shorter time window might also result in comparable perfusion data to [^18^F]FDG PET imaging.

While most of studies evaluated the usefulness of early-phase amyloid PET acquisitions for patients with AD due to its relationship with Aβ, cerebral patterns of hypometabolic regions in [^18^F]FDG PET images can also differentiate AD from other neurodegenerative diseases such as frontotemporal dementia (FTD), dementia with Lewy Bodies, and Parkinson’s disease (PD) [37,38]. Kuo et al. [24] compared early-phase [^18^F]FBP PET images of patients with primary progressive aphasia (PPA) to AD and healthy controls, and Asghar et al. [25] based their analysis specifically on the differentiation between behavioral variant FTD (bvFTD) from AD and cognitively normal elderly. Lastly, Yoo et al. [39] analyzed early-phase [^18^F]FBB PET in patients with PD.

The aim of this study is to quantitatively evaluate the perfusion information of static brain amyloid PET images obtained in the first minute after radiotracer injection, hereafter called FMFs (first-minute-frame), with ^18^F- labeled radiotracers, compared to the cerebral metabolism as measured in [^18^F]FDG PET images. Therefore, a common time frame for early-phase images of the three ^18^F-labeled amyloid radiotracers is tested, which also does not require a dynamic acquisition protocol. Additionally, we present, to the best of our knowledge, the first study evaluating early acquisitions with [^18^F]FMM in comparison to [^18^F]FDG PET. The correlation between FMFs and [^18^F]FDG PET images is evaluated in a retrospective cohort of patients with cognitive impairment or dementia. Different interpatient and intrapatient correlation tests between regional quantitative values of radiotracer uptake are performed to quantitatively assess the comparability of both images.

## 2. Materials and Methods

### 2.1. Patients

All patients that were transferred for suspected neurocognitive disease between February 2016 and July 2019 to the Department of Nuclear Medicine of the Hospital Universitario 12 de Octubre, Madrid, Spain, for amyloid and [^18^F]FDG PET imaging are included in the retrospective study cohort. Both amyloid positive (Aβ+) and amyloid negative (Aβ−) cases are included as demonstrated by visual interpretation of their latest amyloid PET scans. Cases without an FMF are excluded, as well as those with a diagnosis of cerebral amyloid angiopathy.

### 2.2. PET Imaging

#### 2.2.1. FMF Imaging

FMFs were acquired based on [^18^F]FBP, [^18^F]FMM, or [^18^F]FBB on a Siemens Biograph 6 True Point PET/CT scanner (Siemens Healthcare, Erlangen, Germany) and doses of 394 ± 40 MBq, 196 ± 20 MBq, and 259 ± 61 MBq, respectively, were administered intravenously. The image acquisition was performed immediately post injection (p.i.) and a static PET scan was recorded for 1 min. Image reconstruction followed the standard amyloid PET procedure defined in the technical details of the different radiotracers. A point spread function (PSF) reconstruction algorithm was used (3 iterations, 21 subsets, all-pass filter) and the images were corrected for attenuation with a low-dose CT scan. Scatter and random correction were also performed. The reconstructed images had a matrix size of 168×168 and voxel size of 4.0728 × 4.0728 × 5 mm^3^.

#### 2.2.2. [^18^F]FDG PET Imaging

All subjects underwent, apart from the acquisition of early-phase amyloid PET images, a standard [^18^F]FDG PET imaging protocol. [^18^F]FDG PET scans were acquired on a Siemens Biograph 6 True Point PET/CT scanner (Siemens Healthcare, Erlangen, Germany). PET scans were recorded 28–70 min after the injection of 245 ± 86 MBq of [^18^F]FDG. Scan duration was 10 min. The images were reconstructed with a PSF algorithm (6 iterations, 16 subsets; Gaussian filter: 3 mm). Attenuation correction based on a low-dose CT scan and random and scatter correction were performed. The reconstructed images had a matrix size of 336 × 336 and voxel size of 1.0182 × 1.0812 × 3 mm^3^. [^18^F]FDG PET images were acquired between 2 and 334 days prior to FMFs.

### 2.3. Image Analysis

All images are preprocessed using Statistical Parametric Mapping 12 (SPM12) (Wellcome Centre for Human Neuroimaging, University College London, London, United Kingdom) [40]. First, a manual orientation by rotating the images and setting the origin of the native space is performed. Then, the images are normalized to a standard space defined by the Montreal Neurological Institute (MNI) using the corresponding CT images as an anatomical reference as described in [41], resulting in images with a matrix size of 91 × 109 × 91 and voxel size of 2 × 2 × 2 mm^3^. The spatially normalized PET images are smoothed with an 8-mm full width at half-maximum Gaussian kernel. The Automatic Anatomical Labelling (AAL2) atlas is then used to segment a total of 8 volumes of interest (VOI) per brain hemisphere based on 74 cortical and subcortical brain regions (specified in Table S1 of the Supplementary Material) [42,43]. These VOIs correspond to the frontal, occipital, parietal, and temporal lobes, and the anterior (ACC) and posterior cingulate cortices (PCC). The 7th region represents the precuneus, which is analyzed separately as it is one of the core regions in typical AD and its variants (except logopenic) [44,45]. Lastly, we also included the striatum in our study. Mean image intensities (activity concentration, Bq/mL) of each region are extracted and normalized to the mean intensity of a reference region using a custom MATLAB script. The resulting ratios are denominated SUV ratios (SUVR) as they are mathematically equivalent. In this study, the atlas regions corresponding to the grey matter part of the cerebellum is used as a reference for all images as it has been demonstrated that it is not affected in AD [46].

### 2.4. Statistical Analysis

Quantitative variables are represented as mean ± standard deviation (SD). FMFs and [^18^F]FDG images are compared by Pearson’s correlation analysis. Pearson’s correlation coefficients (*r*) are calculated based on the SUVRs of the regions defined by the AAL2 atlas and of the grouped regions. First, the correlation between the intensity profiles of FMFs and [^18^F]FDG is calculated, computed by averaging the regional SUVRs of the corresponding subcohort (study cohort, dividing by radiotracer and amyloid state). Intrapatient correlations are calculated to evaluate the comparability of the images and assess whether FMFs present the same information as their corresponding [^18^F]FDG images. Differences between radiotracers are evaluated by the Kruskal–Wallis tests and subcohort-specific differences between Aβ+ and Aβ− groups by Mann–Whitney U-tests. Additionally, interpatient correlations of regional SUVRs are calculated. Correlations are computed in the whole study group, as well as divided by the amyloid state and used amyloid radiotracer to account for the effect these might have on the FMFs. Differences of regional correlation coefficients are evaluated by Friedman’s test and post hoc pair-wise tests with Bonferroni adjusted α values. The Wilcoxon signed-rank test is used to evaluate subcohort-specific differences of regional correlation coefficients between Aβ+ and Aβ− groups. Using the Benjamini–Hochberg procedure, false discovery rate (FDR) is controlled at level 0.05 where necessary to adjust for multiple comparisons. Statistical analyses are performed in MATLAB R2019a (The MathWorks, Inc., Natick, MA, USA) and SPSS software version 28.00 (IBM Corp., Armonk, NY, USA).

## 3. Results

### 3.1. Demographics and Image Data

A total of 60 patients (age 66.27 ± 8.26 years, female: 31) with available FMF and [^18^F]FDG PET scans comprised the final retrospective study cohort. The standard late-phase amyloid PET scans of 25 patients were visually diagnosed as Aβ+ and 35 as Aβ−. Fourteen subjects were clinically classified as having mild cognitive impairment (MCI) and 36 patients with dementia, in which 16 of them classified as having dementia probably due to AD and 20 with dementia possibly due to AD, based on [47]. The remaining 10 patients were classified as having other dementia, with 7 patients as a differential diagnosis between FTD and 3 patients with unclassifiable primary progressive aphasia (ucPPA). All diagnoses were extracted from clinical records and reviewed for this study by neurologists and nuclear medicine specialists. Patient demographics of the study cohort, as well as of the subcohorts based on the used radiotracer, are summarized in Table 1.

**Figure 1:**
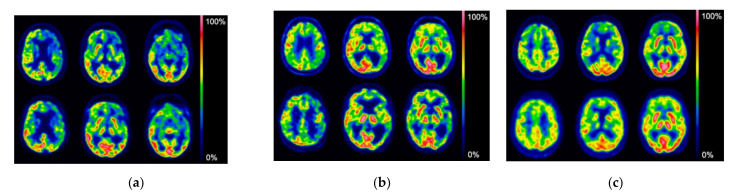
Three representative axial brain slices of [^18^F]FDG PET images (upper row) and their corresponding FMF for each Aβ radiotracer (lower row) are shown using a rainbow color scale in the Xeleris processing system (GE Healthcare, Chicago, IL, USA). (**a**) [^18^F]FBB FMF: 68-year-old male with mild dementia and non-fluent variant PPA (nfvPPA), suggests FTD. [^18^F]FDG PET and [^18^F]FBP FMF show severe left frontal involvement and left parietal and temporal involvement. The amyloid PET scan was negative. (**b**) [^18^F]FMM FMF: 73-year-old male diagnosed with possible AD and amnestic long-term cognitive decline, and as an amnesic, with an MRI with atrophy, and a history of alcoholism. [^18^F]FDG PET and [^18^F]FMM FMF show severe left parietal and frontotemporal involvement. The amyloid PET scan was positive. (**c**) [^18^F]FBP FMF: 69-year-old woman diagnosed with probable AD due to hippocampal amnestic deterioration. Both the [^18^F]FDG PET and [^18^F]FBB FMF images show slight bilateral parietal involvement which is greater on the right side and slight right frontal involvement. The amyloid PET scan was positive.

A visual comparison of [^18^F]FDG brain PET images and their corresponding FMFs for each amyloid radiotracer is exemplified in Figure 1. Overall, [^18^F]FDG PET and FMFs present the same patterns of normal and reduced metabolism.

### 3.2. SUVR Analysis

Regional SUVRs are summarized in Table 2. Through analyzing by specific regions, it can be observed that usually the lowest SUVRs are present in the temporal VOI and striatum. This hypoperfusion/hypometabolism in the temporal region is consistent with patterns found in patients of AD and in the majority of diagnoses of the patients comprising the study groups [37,38]. In contrast, the occipital VOI shows the highest mean SUVR in all subcohorts, which could be attributed mainly to the usual visual activity during the PET scan. In all cases, regional SUVRs are lower in FMFs than [^18^F]FDG PET.

**Figure 2:**
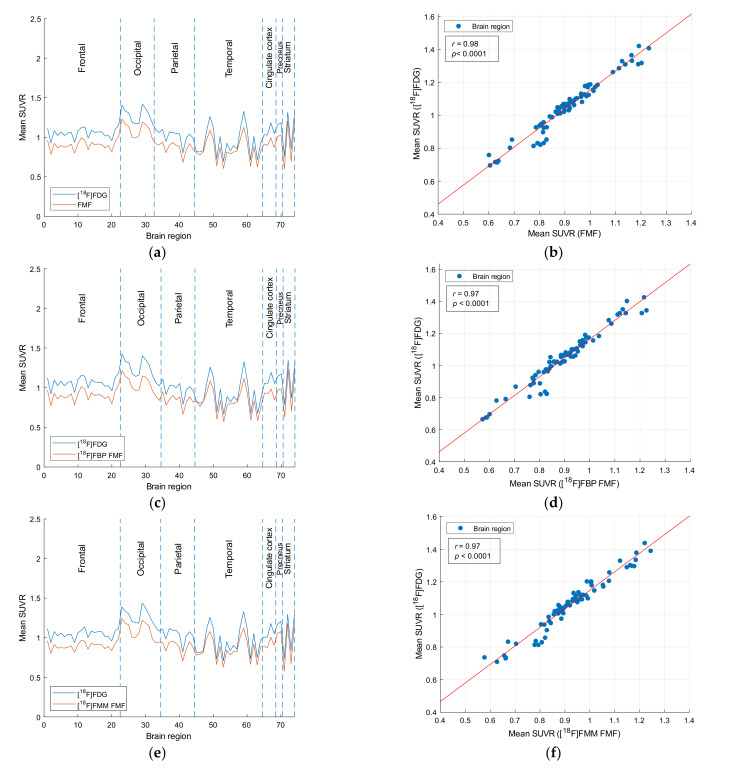

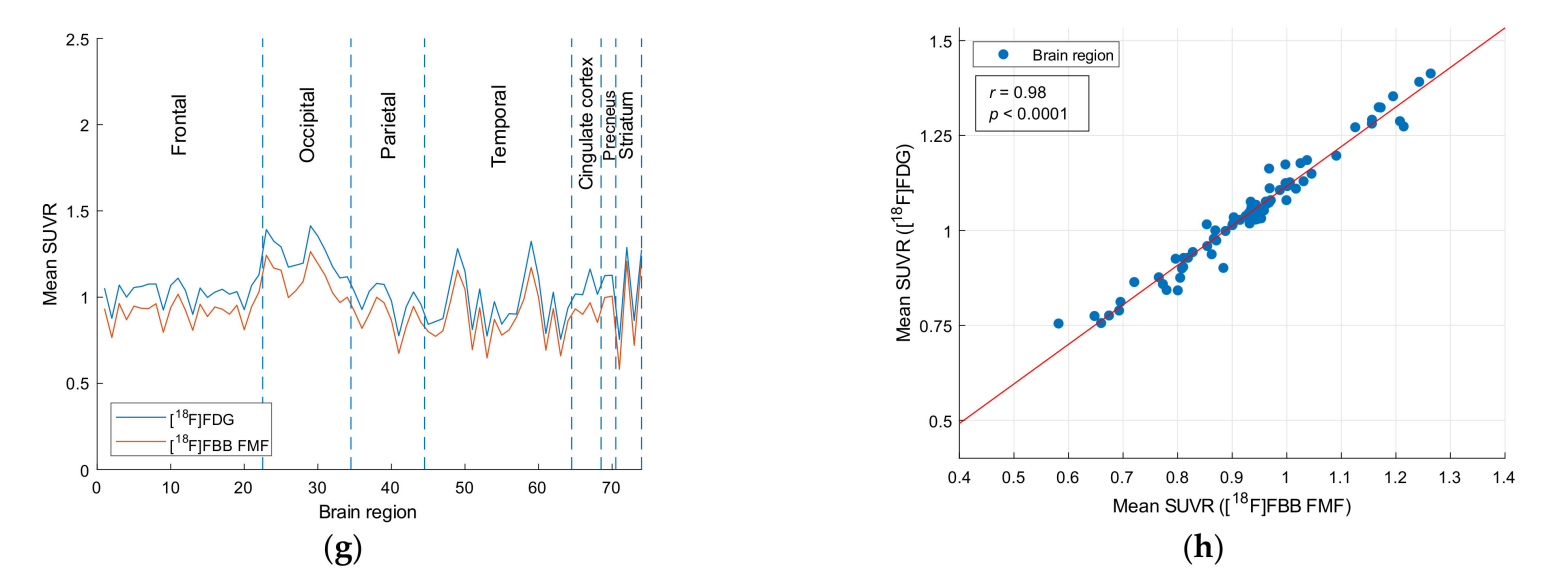
Intensity profiles and correlation charts with least squares line of the mean SUVRs of FMFs and [^18^F]FDG PET. (**a**,**b**) Study cohort; (**c**,**d**) [^18^F]FBP FMFs; (**e**,**f**) [^18^F]FMM FMFs; and (**g**,**h**) [^18^F]FBB FMFs. Brain regions are sorted as specified in Table S1 of the Supplementary Material (left hemisphere followed by right hemisphere).

This trend can also be seen in the corresponding intensity profiles which include the 74 brain regions that define the grouped VOIs (Figure 2). Moreover, the two profiles are strongly correlated as can be seen in the correlation charts of the intensity profiles in Figure 2. The correlation coefficient is *r* = 0.98 (*p* < 0.001) for all patients and in the [^18^F]FBB subcohort, and *r* = 0.97 (*p* < 0.001) in the [^18^F]FBP and [^18^F]FMM subcohorts. Correlation coefficients between intensity profiles in Aβ+ and Aβ− patients range from *r* = 0.96 (*p* < 0.001) to *r* = 0.98 (*p* < 0.001). Regional SUVRs, intensity profile graphs, and correlation charts with the respective correlation coefficients of the Aβ+ and Aβ− subcohorts can be found in Section S2.1 of the Supplementary Materials.

### 3.3. Correlation Analyses

#### 3.3.1. Intrapatient Correlation Analysis

Intrapatient correlation coefficients are summarized in Table 3. In the study cohort and subcohorts, FMFs and [^18^F]FDG PET are strongly correlated. The highest mean intrapatient correlation coefficient is observed when comparing [^18^F]FMM FMFs to their corresponding [^18^F]FDG PET in Aβ− patients (*r* = 0.95 ± 0.05). In contrast, the lowest mean intrapatient correlation coefficient is obtained in the [^18^F]FBP subcohort in Aβ+ patients (*r* = 0.91 ± 0.07). No statistically significant differences of intrapatient correlation coefficients between radiotracers can be found (all patients: *p* = 0.456, Aβ+: *p* = 0.771 and Aβ−: *p* = 0.628). Additionally, intrapatient correlation coefficients are not significantly different between Aβ+ and Aβ− patients (study cohort: *p* = 0.058, [^18^F]FBP: *p* = 0.354, [^18^F]FMM: *p* = 0.093, and [^18^F]FBB: *p* = 0.827).

#### 3.3.2. Interpatient Correlation Analysis

Regional interpatient correlation coefficients of the grouped VOIs are summarized in Table 4. The highest statistically significant correlation is *r* = −1.00 (*p* < 0.001), observed in the left occipital of Aβ+ patients in the [^18^F]FBB subcohort. However, given the small number of patients (N = 3), we do not consider this result representative. In the [^18^F]FBB subcohort, the correlation coefficients for the subcohort (mean *r* = 0.77 ± 0.26), and after separating it by amyloid state, are naturally lower or often not statistically significant or representative. Comparing the three first groups (study cohort, [^18^F]FBP subcohort, and [^18^F]FMM subcohort) and without separating by amyloid state, the highest correlation coefficient is observed in the right temporal VOI of the [^18^F]FBP subcohort (*r* = 0.94 (*p* < 0.001)). Strong and statistically significant correlations are observed in all brain regions for the study cohort (mean *r* = 0.82 ± 0.06) as well as for the [^18^F]FBP (mean *r* = 0.86 ± 0.08) and [^18^F]FMM (mean *r* = 0.78 ± 0.10) subcohorts. No statistically significant differences are observed between the regional correlation coefficients of all patients of the three radiotracers (*p* = 0.126). 

Separated by amyloid state, regional correlation coefficients are higher in Aβ+ patients than Aβ− patients in the study cohort (Aβ+: mean *r* = 0.80 ± 0.08 and Aβ−: mean *r* = 0.78 ± 0.12, *p* = 0.642), [^18^F]FBP subcohort (Aβ+: mean *r* = 0.85 ± 0.09 and Aβ−: mean *r* = 0.79 ± 0.14, *p* = 0.501), and [^18^F]FBB subcohort (Aβ+: mean *r* = 0.94 ± 0.10 and Aβ−: mean *r* = 0.16 ± 0.84, *p* = 0.005), while they are lower in the [^18^F]FMM subcohort (Aβ+: mean *r* = 0.62 ± 0.38 and Aβ−: mean *r* = 0.82 ± 0.08, *p* = 0.020). Statistically significant differences of the regional correlation coefficients between radiotracers are found only in Aβ+ patients (*p* = 0.001) with differences between the [^18^F]FBB subcohort and both the [^18^F]FBP and [^18^F]FMM subcohorts found in post hoc analyses (*p* = 0.008 and *p* = 0.002, respectively). However, no differences are observed in Aβ− patients (*p* = 0.099).

Lastly, the mean interpatient correlation coefficients of the 74 brain regions comprising the grouped VOIs and used for the construction of the intensity profiles are summarized in Table 5. The highest mean correlation coefficient is obtained in the [^18^F]FBP subcohort independently of the amyloid state (mean *r* = 0.82 ± 0.12). While the lowest mean correlation coefficient is found in the [^18^F]FBB subcohort in Aβ− patients (mean *r* = 0.33 ± 0.59), these are not statistically significant or representative due to the low sample size (N = 3). Statistically significant differences of correlation coefficients between radiotracers are observed in all three amyloid state subgroups (*p* < 0.001). Post hoc analyses showed significant differences between the [^18^F]FBP subcohort and both the [^18^F]FMM and [^18^F]FBB subcohorts for all patients (*p* < 0.001), and between the [^18^F]FBB subcohort and both the [^18^F]FBP and [^18^F]FMM subcohorts for Aβ+ and Aβ− patients (*p* < 0.001). Statistically significant differences of correlation coefficients between Aβ+ and Aβ− patients are found in the [^18^F]FMM and [^18^F]FBB subcohorts (both *p* < 0.001) but not in the study cohort (*p* = 0.790) or the [^18^F]FBP subcohort (*p* = 0.230).

## 4. Discussion

The correlation between the FMF, static brain PET images corresponding to the first p.i. minute frame of a ^18^F-labeled Aβ-binding radiotracer, and [^18^F]FDG PET images is investigated in this study. Previously, dynamic PET images based on Aβ-binding radiotracers were acquired during the first 10 min p.i. and perfusion-like early-phase amyloid PET images were then computed based on the dynamic data and radiotracer-specific time frames usually by averaging or summing the specific frames [10,11,12,13,14,15,17,18,20,21,22,23,24,25,26,28,29,33]. The FMF is therefore studied as an alternative method more widely available with a common time frame for the three ^18^F-labeled Aβ-binding radiotracers, which is also easier to acquire and has higher usability in routine clinical practice. Additionally, the possibility to study both neurodegeneration and Aβ deposition with one radiotracer injection and in one scan session would be clinically highly beneficial.

In this study, FMFs were acquired with the three approved and commercially available ^18^F-labeled amyloid tracers, namely [^18^F]FBP, [^18^F]FBB, and [^18^F]FMM [8]. Whereas early-phase amyloid PET images have been evaluated in various studies as a surrogate for cerebral metabolism using [^18^F]FBP [22,23,24,25,26,27] and [^18^F]FBB [19,28,29,30,31], no study comparing early-phase amyloid PET acquisitions with [^18^F]FMM to [^18^F]FDG PET images was found during the writing of this manuscript. However, the optimal time windows for the acquisition of dual-phase [^18^F]FMM PET images are being investigated [32] in addition to its perfusion information [33]. The aim was also to study the comparability of the FMF to [^18^F]FDG PET images independently of the cognitive stage using a study cohort with patients situated in a broad spectrum. Subjects from routine clinical practice with different preliminary diagnoses classified as probable AD or possible AD as defined in [47], differential diagnosis with FTD, and other types of dementia were included. However, future studies could focus on specific conditions as it has been done for PPA [24] and FTD [25], or other diseases such as PD [39].

SUVR analyses revealed that while [^18^F]FDG PET images present higher mean SUVRs in all regions, the intensity profiles show high similarity and correlation up to *r* = 0.98 (*p* < 0.001) (Figure 2). This is consistent with previous studies comparing early-phase [^11^C]PiB [11,15,20], [^18^F]FBP [22], or [^18^F]FBB [28,29] to [^18^F]FDG PET images. Segovia et al. [29] specifically analyzed the intensity profile of early-phase [^18^F]FBB and [^18^F]FDG PET images and found similar mean regional intensity values for both images. Lin et al. [23] evaluated global SUVR values of early-phase [^18^F]FBP PET images of patients with different stages of MCI and AD. The authors report a progressive decrease of the global SUVR for more advanced stages, indicating more extensive regions of hypoperfusion. This is similar to [^18^F]FDG PET images and decrease of the global SUVR based on the extension of the hypometabolic regions.

The high comparability of the FMF and the patients’ corresponding [^18^F]FDG PET images is confirmed in the intrapatient analysis. The mean correlation coefficients of the different studied cohorts reach as high as *r* = 0.95 ± 0.05. This is in agreement with other studies evaluating within-subject correlation between early-phase amyloid and [^18^F]FDG PET images [10,23,25]. In all cases, the intrapatient correlation coefficients are comparable or higher than those reported previously.

Visual analysis of [^18^F]FDG brain PET images is generally based on the evaluation of radiotracer uptake patterns in anatomo-functional cortical regions and meta VOIs are created from more detailed brain parcels for regional quantitative analysis [48]. The majority of studies comparing early-phase amyloid and [^18^F]FDG PET images are based on a meta-VOI analysis. Regional interpatient correlation analyses of the 16 grouped VOIs of this study result in overall strong correlation values between FMFs and [^18^F]FDG PET images. The parietal, posterior cingulate cortex, precuneus, and temporal VOIs are of special interest due to being specific core regions for the diagnosis of AD, but which are also affected in other types of dementia evaluated in routine clinical practice [37,38,44]. Correlation is usually higher than *r* = 0.80 in these regions between FMFs and [^18^F]FDG PET with a few exceptions. In the case of early-phase acquisitions with [^18^F]FBP, Hsiao et al. [23] reported correlation coefficients of up to *r* = 0.93 in the superior temporal region, compared to *r* = 0.94 in the right temporal VOI in the corresponding subcohort in our study. Other studies evaluated imaging characteristics of early-phase [^18^F]FBP PET images specifically in patients with PPA [24] or bvFTD [25] to demonstrate the viability of early-phase brain amyloid PET images in neurodegenerative diseases other than purely AD. Evaluating FMFs acquired with different Aβ-binding tracers, the correlation values obtained in our study are comparable, ranging from *r* = 0.71 in the left occipital VOI to *r* = 0.92 in the striatum. Tiepolt et al. [19] analyzed a mixed study group with early-phase [^18^F]FBB and [^11^C]PiB PET images, yielding correlation values ranging from *r* = 0.61 in the frontal region to *r* = 0.79 in the posterior cingulate cortex. In another study employing [^18^F]FBB, Daerr et al. [19] obtained correlation values between *r* = 0.59 and *r* = 0.86 with a time frame of 0–5 min and cerebellar normalization. The authors achieved higher correlation values with global mean normalization and separating Aβ−positive from negative cases. While not directly comparable to our results due to the small sample size of the [^18^F]FBB subcohort, these values are comparable. Moreover, a slight increase of correlation coefficients in Aβ+ patients compared to Aβ− patients can be observed in our study despite not showing statistically significant differences, similarly to the results of Daerr et al. However, a statistically significant difference in the [^18^F]FMM subcohort indicates higher correlation coefficients in the Aβ− group. Additionally, Son et al. [31] obtained correlation coefficients reaching *r* = 0.8752 in the left temporal lobe, comparing static 5-min early [^18^F]FBB PET images to [^18^F]FDG PET. In our study, the [^18^F]FBB FMF shows a correlation of *r* = 0.91 in the left temporal VOI to [^18^F]FDG PET. Son et al. also reported higher SUVRs in [^18^F]FDG PET compared to early-phase [^18^F]FBB PET, which matches the intensity profiles shown by the FMF. 

In this study, smaller brain regions are also analyzed to evaluate the correlation between the images at a more detailed level. A detailed brain parcellation and subsequent correlation analysis was conducted by Segovia et al. [29] using early-phase [^18^F]FBB and [^18^F]FDG PET images. The authors obtained the highest correlation value in the caudate but only the caudate and right Heschl gyrus are shown to present correlation coefficients above *r* = 0.5. In general, correlation coefficients for AAL2-defined regions are higher in our study, especially considering the different sample sizes (mean in the study cohort *r* = 0.80 ± 0.09). While we believe that the statistically significant differences found between the [^18^F]FBB subcohort and the others is due to its small sample size and are thus not representative, [^18^F]FBP also showed higher correlation coefficients in these regions than in the [^18^F]FMM subcohort without considering amyloid state.

Lastly, of special interest are the results of the [^18^F]FMM subcohort. In our study, the correlation between [^18^F]FMM and [^18^F]FDG PET is comparable to the correlation in the [^18^F]FBP subcohort, with the same number of patients in each. The [^18^F]FMM FMF shows a similar intensity profile to [^18^F]FDG PET (*r* = 0.97, *p* < 0.001), strong intrapatient correlations (*r* = 0.94 ± 0.05), and regional interpatient correlations (up to *r* = 0.93, *p* < 0.001). While the dual-time protocol and perfusion information of [^18^F]FMM has been studied [32,33], the correlation of early-phase [^18^F]FMM images to [^18^F]FDG was not analyzed before and therefore no comparisons of our results in this subcohort to previous studies could be made.

In this study, four main limitations are identified. After a preliminary visual comparison of FMFs, a static amyloid PET image of the first minute p.i. was selected for quantitative analysis and possible alternative to [^18^F]FDG PET and dynamic early-phase amyloid PET images. However, in several of the above-mentioned studies, different time frames for the acquisition of perfusion amyloid PET images were identified. Therefore, static amyloid PET images of the first 10 min will additionally be evaluated, comparing them to the FMF and [^18^F]FDG PET in future studies. Moreover, it should be noted that it would be desirable for FMFs to be acquired a standardized 5–10 s after the injection of the radiotracer to avoid a loss of initial counts in some images. It can be assumed that the quantitative correlation between FMF and [^18^F]FDG PET images would be slightly higher if there is optimal coordination between injection and acquisition time. 

Regarding the subject cohort, it is only composed of patients with various degrees of cognitive decline from MCI to AD and no cognitively normal subjects have been included. The main aim of this study is to assess the viability of the FMF for the diagnosis and evaluation of neurodegenerative diseases, as it is one of the main clinical applications of brain [^18^F]FDG PET in routine clinical practice. A healthy control group would have been of interest to complete the analysis and validation of the FMF method. Therefore, in future studies, the comparability of the FMF to [^18^F]FDG will be studied in cognitively normal subjects. Another limitation of the study group concerns the absence of FMFs acquired with [^11^C]PiB and the especially the low number of FMFs with [^18^F]FBB, which result in not statistically significant or not representative results. Finally, the time between the FMF acquisition and the corresponding [^18^F]FDG PET images was not synchronized, with scans acquired between 2 and 334 days apart. While changes of the perfusion/metabolism due to neurodegenerative diseases are slow (years), they might introduce slight differences in uptake patterns in cases where the dates of the image acquisitions are further apart and therefore reduce the correlation coefficients.

Even though some of the presented results are conditioned by the available study cohort, the potential of the FMF and its comparability to [^18^F]FDG PET images is demonstrated. It is shown that in most cases the correlation between the images is not significantly affected by the radiotracer or amyloid state. Moreover, early acquisitions with [^18^F]FMM demonstrate to offer the same comparable information to [^18^F]FDG as [^18^F]FBP and [^18^F]FBB. Further retrospective and prospective studies will be conducted to confirm the findings and optimize the acquisition protocol. While in this study all images were acquired using a PET/CT scanner, it would be interesting to evaluate the utility of FMFs with PET/MRI. Additionally, studies evaluating the visual comparability will be performed to validate the clinical usefulness of the FMF as a diagnostic tool of neurodegeneration.

## 5. Conclusions

The FMF shows quantitative similarities to [^18^F]FDG brain PET images, with strong correlations using [^18^F]FBP, [^18^F]FMM, or [^18^F]FBB to [^18^F]FDG PET images in a group of patients with different degrees of cognitive impairment. Additionally, [^18^F]FMM FMFs show a strong correlation to [^18^F]FDG PET, whose early acquisitions are evaluated quantitatively and compared to metabolic PET for the first time in this study. The FMF protocol is a viable static PET alternative to dynamic early-phase brain amyloid PET imaging in clinical practice, providing information of neuronal injury similar to that shown by [^18^F]FDG PET imaging.

## Figures and Tables

**Table 1:** Study cohort demographics. Classification in possible and probable AD was based on [47].

	N	Age (Years ± SD)	Sex (m/f)	Amyloid State (Aβ+/Aβ−)	MCI	Possible AD	Probable AD	Other Dementias
Study cohort	60	66.27 ± 8.26	29/31	25/35	14	20	16	10
[^18^F]FBP FMF	27	66.33 ± 7.92	17/10	12/15	3	9	8	6
[^18^F]FMM FMF	27	65.15 ± 9.10	10/17	10/17	8	9	8	3
[^18^F]FBB FMF	6	71.00 ± 0.00	2/4	3/3	3	2	0	1

**Table 2:** Regional SUVRs of FMFs and [^18^F]FDG PET.

Brain Region		SUVR (Mean ± SD)							
		Study Cohort		[^18^F]FBP Subcohort		[^18^F]FMM Subcohort		[^18^F]FBB Subcohort	
		FMF	[^18^F]FDG	FMF	[^18^F]FDG	FMF	[^18^F]FDG	FMF	[^18^F]FDG
Frontal	L	0.88 ± 0.11	1.03 ± 0.16	0.87 ± 0.09	1.03 ± 0.15	0.88 ± 0.14	1.02 ± 0.19	0.88 ± 0.12	1.01 ± 0.13
	R	0.90 ± 0.08	1.05 ± 0.13	0.90 ± 0.08	1.06 ± 0.13	0.90 ± 0.09	1.05 ± 0.14	0.89 ± 0.11	1.00 ± 0.12
Occipital	L	1.09 ± 0.10	1.27 ± 0.15	1.07 ± 0.11	1.26 ± 0.14	1.11 ± 0.09	1.27 ± 0.16	1.12 ± 0.11	1.26 ± 0.15
	R	1.05 ± 0.11	1.24 ± 0.14	1.02 ± 0.12	1.22 ± 0.14	1.08 ± 0.10	1.26 ± 0.14	1.10 ± 0.06	1.25 ± 0.09
Parietal	L	0.90 ± 0.09	1.05 ± 0.15	0.88 ± 0.10	1.03 ± 0.16	0.92 ± 0.08	1.08 ± 0.14	0.91 ± 0.11	1.02 ± 0.13
	R	0.84 ± 0.09	0.98 ± 0.13	0.82 ± 0.10	0.97 ± 0.14	0.85 ± 0.08	1.00 ± 0.12	0.84 ± 0.08	0.94 ± 0.09
Temporal	L	0.85 ± 0.12	0.96 ± 0.16	0.83 ± 0.12	0.94 ± 0.16	0.87 ± 0.12	0.98 ± 0.16	0.90 ± 0.13	1.00 ± 0.13
	R	0.86 ± 0.11	0.97 ± 0.15	0.82 ± 0.13	0.94 ± 0.17	0.89 ± 0.09	1.00 ± 0.14	0.89 ± 0.09	0.99 ± 0.10
ACC	L	0.92 ± 0.16	1.03 ± 0.17	0.93 ± 0.10	1.06 ± 0.12	0.89 ± 0.20	1.01 ± 0.22	0.93 ± 0.14	1.02 ± 0.11
	R	0.90 ± 0.13	1.03 ± 0.15	0.92 ± 0.08	1.06 ± 0.10	0.87 ± 0.17	1.01 ± 0.19	0.90 ± 0.10	1.01 ± 0.09
PCC	L	0.99 ± 0.14	1.18 ± 0.22	0.98 ± 0.13	1.19 ± 0.23	1.01 ± 0.14	1.18 ± 0.21	0.97 ± 0.20	1.16 ± 0.24
	R	0.87 ± 0.14	1.05 ± 0.20	0.84 ± 0.16	1.05 ± 0.22	0.90 ± 0.14	1.05 ± 0.19	0.85 ± 0.10	1.02 ± 0.17
Precuneus	L	1.01 ± 0.11	1.16 ± 0.18	0.97 ± 0.12	1.15 ± 0.20	1.05 ± 0.10	1.17 ± 0.17	1.00 ± 0.12	1.12 ± 0.14
	R	1.03 ± 0.13	1.18 ± 0.18	0.99 ± 0.14	1.17 ± 0.22	1.08 ± 0.11	1.21 ± 0.16	1.01 ± 0.12	1.13 ± 0.10
Striatum	L	0.60 ± 0.18	0.76 ± 0.21	0.63 ± 0.14	0.78 ± 0.19	0.58 ± 0.22	0.74 ± 0.25	0.58 ± 0.15	0.75 ± 0.19
	R	0.69 ± 0.17	0.85 ± 0.20	0.70 ± 0.16	0.87 ± 0.18	0.67 ± 0.19	0.83 ± 0.23	0.72 ± 0.14	0.86 ± 0.18

**Table 3:** Mean ± SD intrapatient correlation coefficients of FMFs and [^18^F]FDG PET.

Subcohort	All Patients	Aβ+	Aβ−
Study cohort	0.93 ± 0.05	0.92 ± 0.05	0.94 ± 0.05
[^18^F]FBP FMF	0.92 ± 0.06	0.91 ± 0.07	0.92 ± 0.06
[^18^F]FMM FMF	0.94 ± 0.05	0.92 ± 0.04	0.95 ± 0.05
[^18^F]FBB FMF	0.94 ± 0.04	0.94 ± 0.04	0.94 ± 0.05

**Table 4:** Regional interpatient correlation coefficients of FMF and [^18^F]FDG PET. * statistically significant FDR-adjusted *p*-value.

Brain Region		Pearson’s Correlation Coefficient *r*											
		Study Cohort			[^18^F]FBP Subcohort			[^18^F]FMM Subcohort			[^18^F]FBB Subcohort		
		All Patients	Aβ+	Aβ−	All Patients	Aβ+	Aβ−	All Patients	Aβ+	Aβ−	All Patients	Aβ+	Aβ−
Frontal	L	0.83 *	0.82 *	0.84 *	0.82 *	0.73 *	0.87 *	0.84 *	0.88 *	0.88 *	0.77	0.99	0.40
	R	0.76 *	0.70 *	0.78 *	0.78 *	0.69 *	0.83 *	0.76 *	0.62	0.83*	0.75	0.91	0.49
Occipital	L	0.71 *	0.74 *	0.57 *	0.82 *	0.89 *	0.49	0.65 *	0.38	0.74 *	0.63	0.99	−1.00 *
	R	0.73 *	0.76 *	0.69 *	0.89 *	0.95 *	0.78 *	0.62 *	−0.24	0.76 *	−0.12	0.92	−0.97
Parietal	L	0.78 *	0.77 *	0.58 *	0.84 *	0.87 *	0.51	0.70 *	0.48	0.75 *	0.77	1.00	−0.97
	R	0.80 *	0.63 *	0.75 *	0.89 *	0.86 *	0.84 *	0.69 *	−0.22	0.79 *	0.69	0.99	−0.87
Temporal	L	0.88 *	0.89 *	0.86 *	0.92 *	0.91 *	0.77 *	0.85 *	0.94 *	0.90 *	0.91 *	0.98	0.61
	R	0.88 *	0.87 *	0.86 *	0.94 *	0.94 *	0.94 *	0.78 *	0.72 *	0.81 *	0.75	0.56	0.69
ACC	L	0.86 *	0.82 *	0.89 *	0.76 *	0.83 *	0.79 *	0.89 *	0.81 *	0.92 *	0.82 *	0.99	0.95
	R	0.83 *	0.78 *	0.89 *	0.65 *	0.70 *	0.74 *	0.87 *	0.86 *	0.92 *	0.93 *	0.84	0.98
PCC	L	0.81 *	0.81 *	0.76 *	0.80 *	0.71 *	0.84 *	0.81 *	0.89 *	0.72 *	0.91 *	0.98	0.89
	R	0.86 *	0.84 *	0.84 *	0.91 *	0.87 *	0.94 *	0.84 *	0.87 *	0.80 *	0.90 *	0.89	0.94
Precuneus	L	0.79 *	0.75 *	0.64 *	0.91 *	0.92 *	0.64 *	0.70 *	0.65	0.77 *	0.83	1.00	−0.99
	R	0.79 *	0.72 *	0.70 *	0.92 *	0.90 *	0.81 *	0.65 *	0.41	0.75 *	0.70	0.98	−0.37
Striatum	L	0.92 *	0.91 *	0.93 *	0.91 *	0.86 *	0.96 *	0.83 *	0.93 *	0.93 *	0.99 *	1.00	0.95
	R	0.92 *	0.93 *	0.92 *	0.91 *	0.91 *	0.94 *	0.93 *	0.95 *	0.91 *	0.91 *	0.98	0.77

**Table 5:** Mean ± SD interpatient correlation coefficients of FMFs and [^18^F]FDG PET of individual brain regions.

Subcohort	All Patients	Aβ+	Aβ−
Study cohort	0.80 ± 0.09	0.78 ± 0.11	0.78 ± 0.12
[^18^F]FBP FMF	0.82 ± 0.12	0.81 ± 0.15	0.77 ± 0.18
[^18^F]FMM FMF	0.77 ± 0.12	0.66 ± 0.28	0.81 ± 0.10
[^18^F]FBB FMF	0.72 ± 0.25	0.82 ± 0.39	0.33 ± 0.59

## Data Availability

The data presented in this study are available on request from the corresponding authors. The data is not publicly available due to clinical patient information.

## Supplementary Materials

The following are available online at https://www.mdpi.com/article/10.3390/s21155182/s1: Table S1: AAL2 brain regions that compose the grouped VOIs; Table S2: Regional SUVRs of FMFs and [^18^F]FDG PET of Aβ+ patients; Table S3: Regional SUVRs of FMFs and [^18^F]FDG PET of Aβ− patients; Figure S1: Intensity profiles and correlation charts with least squares line of the mean SUVRs between FMFs and [^18^F]FDG PET of Aβ+ patients; and Figure S2: Intensity profiles and correlation charts with least squares line of the mean SUVRs between FMFs and [^18^F]FDG PET of Aβ− patients.

## References

[1] Jack C.R., Bennett D.A., Blennow K., Carrillo M.C., Dunn B., Haeberlein S.B., Holtzman D.M., Jagust W., Jessen F., Karlawish J. (2018). NIA-AA Research Framework: Toward a biological definition of Alzheimer’s disease. Alzheimer’s Dement..

[2] Chen G.F., Xu T.H., Yan Y., Zhou Y.R., Jiang Y., Melcher K., Xu H.E. (2017). Amyloid beta: Structure, biology and structure-based therapeutic development. Acta Pharmacol. Sin..

[3] Ikonomovic M.D., Klunk W.E., Abrahamson E.E., Mathis C.A., Price J.C., Tsopelas N.D., Lopresti B.J., Ziolko S., Bi W., Paljug W.R. (2008). Post-mortem correlates of in vivo PiB-PET amyloid imaging in a typical case of Alzheimer’s disease. Brain.

[4] Clark C.M., Pontecorvo M.J., Beach T.G., Bedell B.J., Coleman R.E., Doraiswamy P.M., Fleisher A.S., Reiman E.M., Sabbagh M.N., Sadowsky C.H. (2012). Cerebral PET with florbetapir compared with neuropathology at autopsy for detection of neuritic amyloid-β plaques: A prospective cohort study. Lancet Neurol..

[5] Sabri O., Sabbagh M.N., Seibyl J., Barthel H., Akatsu H., Ouchi Y., Senda K., Murayama S., Ishii K., Takao M. (2015). Florbetaben PET imaging to detect amyloid beta plaques in Alzheimer’s disease: Phase 3 study. Alzheimer’s Dement..

[6] Curtis C., Gamez J.E., Singh U., Sadowsky C.H., Villena T., Sabbagh M.N., Beach T.G., Duara R., Fleisher A.S., Frey K.A. (2015). Phase 3 trial of flutemetamol labeled with radioactive fluorine 18 imaging and neuritic plaque density. JAMA Neurol..

[7] Sokoloff L. (1981). Relationships among local functional activity, energy metabolism, and blood flow in the central nervous system. Fed. Proc..

[8] Tiepolt S., Patt M., Aghakhanyan G., Meyer P.M., Hesse S., Barthel H., Sabri O. (2019). Current radiotracers to image neurodegenerative diseases. EJNMMI Radiopharm. Chem..

[9] Meyer P.T., Hellwig S., Amtage F., Rottenburger C., Sahm U., Reuland P., Weber W.A., Hüll M. (2011). Dual-biomarker imaging of regional cerebral amyloid load and neuronal activity in dementia with PET and 11C-Labeled Pittsburgh compound B. J. Nucl. Med..

[10] Rostomian A.H., Madison C., Rabinovici G.D., Jagust W.J. (2011). Early 11C-PIB frames and 18F-FDG PET measures are comparable: A study validated in a cohort of AD and FTLD patients. J. Nucl. Med..

[11] Peretti D.E., Vállez García D., Reesink F.E., van der Goot T., De Deyn P.P., de Jong B.M., Dierckx R.A.J.O., Boellaard R. (2019). Relative cerebral flow from dynamic PIB scans as an alternative for FDG scans in Alzheimer’s disease PET studies. PLoS ONE.

[12] Oliveira F.P.M., Moreira A.P., De Mendonça A., Verdelho A., Xavier C., Barroca D., Rio J., Cardoso E., Cruz Â., Abrunhosa A. (2018). Can 11C-PiB-PET relative delivery R1 or 11C-PiB-PET perfusion replace 18F-FDG-PET in the assessment of brain neurodegeneration?. J. Alzheimer’s Dis..

[13] Ponto L.L.B., Moser D.J., Menda Y., Harlynn E.L., DeVries S.D., Oleson J.J., Magnotta V.A., Schultz S.K. (2019). Early Phase PIB-PET as a Surrogate for Global and Regional Cerebral Blood Flow Measures. J. Neuroimaging.

[14] Forsberg A., Engler H., Blomquist G., Långström B., Nordberg A. (2012). The use of PIB-PET as a dual pathological and functional biomarker in AD. Biochim. Biophys. Acta Mol. Basis Dis..

[15] Fu L., Liu L., Zhang J., Xu B., Fan Y., Tian J. (2014). Comparison of dual-biomarker PIB-PET and dual-tracer PET in AD diagnosis. Eur. Radiol..

[16] Chen Y.J., Rosario B.L., Mowrey W., Laymon C.M., Lu X., Lopez O.L., Klunk W.E., Lopresti B.J., Mathis C.A., Price J.C. (2015). Relative 11C-PiB delivery as a proxy of relative CBF: Quantitative evaluation using single-session 15o-water and 11C-PiB PET. J. Nucl. Med..

[17] Farid K., Hong Y.T., Aigbirhio F.I., Fryer T.D., Menon D.K., Warburton E.A., Baron J.-C. (2015). Early-Phase 11C-PiB PET in Amyloid Angiopathy-Related Symptomatic Cerebral Hemorrhage: Potential Diagnostic Value?. PLoS ONE.

[18] Gietl A.F., Warnock G., Riese F., Kälin A.M., Saake A., Gruber E., Leh S.E., Unschuld P.G., Kuhn F.P., Burger C. (2015). Regional cerebral blood flow estimated by early PiB uptake is reduced in mild cognitive impairment and associated with age in an amyloid-dependent manner. Neurobiol. Aging.

[19] Tiepolt S., Hesse S., Patt M., Luthardt J., Schroeter M.L., Hoffmann K.-T., Weise D., Gertz H.-J., Sabri O., Barthel H. (2016). Early [18F]Florbetaben and [11C]PiB PET images are a surrogate biomarker of neuronal injury in Alzheimer’s disease. Eur. J. Nucl. Med. Mol. Imaging.

[20] Rodriguez-Vieitez E., Carter S.F., Chiotis K., Saint-Aubert L., Leuzy A., Schöll M., Almkvist O., Wall A., Långström B., Nordberg A. (2016). Comparison of early-phase 11C-Deuterium-L-Deprenyl and 11C-Pittsburgh Compound B PET for Assessing Brain Perfusion in Alzheimer Disease. J. Nucl. Med..

[21] Rodriguez-Vieitez E., Leuzy A., Chiotis K., Saint-Aubert L., Wall A., Nordberg A. (2017). Comparability of [18F]THK5317 and [11C] PIB blood flow proxy images with [18F]FDG positron emission tomography in Alzheimer’s disease. J. Cereb. Blood Flow Metab..

[22] Hsiao I.T., Huang C.C., Hsieh C.J., Hsu W.C., Wey S.P., Yen T.C., Kung M.P., Lin K.J. (2012). Correlation of early-phase 18F-florbetapir (AV-45/Amyvid) PET images to FDG images: Preliminary studies. Eur. J. Nucl. Med. Mol. Imaging.

[23] Lin K.J., Hsiao I.T., Hsu J.L., Huang C.C., Huang K.L., Hsieh C.J., Wey S.P., Yen T.C. (2016). Imaging characteristic of dual-phase 18F-florbetapir (AV-45/Amyvid) PET for the concomitant detection of perfusion deficits and beta-amyloid deposition in Alzheimer’s disease and mild cognitive impairment. Eur. J. Nucl. Med. Mol. Imaging.

[24] Kuo H.C., Hsiao I.T., Hsieh C.J., Huang C.Y., Huang K.L., Wai Y.Y., Chuang W.L., Kung M.P., Chu Y.C., Yen T.C. (2017). Dual-phase 18F-florbetapir positron emission tomography in patients with primary progressive aphasia, Alzheimer’s disease, and healthy controls: A preliminary study. J. Formos. Med. Assoc..

[25] Asghar M., Hinz R., Herholz K., Carter S.F., Carter S.F. (2019). Dual-phase [18F] florbetapir in frontotemporal dementia. Eur. J. Nucl. Med. Mol. Imaging.

[26] Ottoy J., Verhaeghe J., Niemantsverdriet E., De Roeck E., Wyffels L., Ceyssens S., Van Broeckhoven C., Engelborghs S., Stroobants S., Staelens S. (2019). 18F-FDG PET, the early phases and the delivery rate of 18F-AV45 PET as proxies of cerebral blood flow in Alzheimer’s disease: Validation against 15O-H2O PET. Alzheimer’s Dement..

[27] Vanhoutte M., Landeau B., Sherif S., De la Sayette V., Dautricourt S., Abbas A., Manrique A., Chocat A., Chetelat G. (2020). Optimization of early-phase florbetapir as a surrogate of FDG-PET in ageing and Alzheimer’s clinical syndrome. Alzheimer’s Dement..

[28] Daerr S., Brendel M., Zach C., Mille E., Schilling D., Zacherl M.J., Bürger K., Danek A., Pogarell O., Schildan A. (2017). Evaluation of early-phase [18F]-florbetaben PET acquisition in clinical routine cases. NeuroImage Clin..

[29] Segovia F., Gómez-Río M., Sánchez-Vañó R., Górriz J.M., Ramírez J., Triviño-Ibáñez E., Carnero-Pardo C., Martínez-Lozano M.D., Sopena-Novales P. (2018). Usefulness of dual-point amyloid PET scans in appropriate use criteria: A multicenter study. J. Alzheimer’s Dis..

[30] Florek L., Tiepolt S., Schroeter M.L., Berrouschot J., Saur D., Hesse S., Jochimsen T., Luthardt J., Sattler B., Patt M. (2018). Dual Time-Point [18F]Florbetaben PET Delivers Dual Biomarker Information in Mild Cognitive Impairment and Alzheimer’s Disease. J. Alzheimer’s Dis..

[31] Son S.H., Kang K., Ko P.-W., Lee H.-W., Lee S.-W., Ahn B.-C., Lee J., Yoon U., Jeong S.Y. (2020). Early-Phase 18F-Florbetaben PET as an Alternative Modality for 18F-FDG PET. Clin. Nucl. Med..

[32] Heeman F., Yaqub M., Lopes Alves I., Heurling K., Berkhof J., Gispert J.D., Bullich S., Foley C., Lammertsma A.A. (2019). Optimized dual-time-window protocols for quantitative [18F]flutemetamol and [18F]florbetaben PET studies. EJNMMI Res..

[33] An Y.S., Yoon J.H., Son S.J., Hong C.H., Lee S.J., Yoon J.K. (2021). Early-phase 18F-FP-CIT and 18F-flutemetamol PET were significantly correlated. Sci. Rep..

[34] Varrone A., Asenbaum S., Borght V.T., Booij J., Nobili F., Någren K., Darcourt J., Kapucu Ö.L., Tatsch K., Bartenstein P. (2009). EANM procedure guidelines for PET brain imaging using [18F]FDG, version 2. Eur. J. Nucl. Med. Mol. Imaging.

[35] Waxman A., Herholz K., Lewis D., Herscovitch P., Minoshima S., Ichise M., Drzezga A., Devous M., Mountz J. (2009). Society of Nuclear Medicine Procedure Guideline for FDG PET Brain Imaging Version 1.0.

[36] Minoshima S., Drzezga A.E., Barthel H., Bohnen N., Djekidel M., Lewis D.H., Mathis C.A., McConathy J., Nordberg A., Sabri O. (2016). SNMMI procedure standard/EANM practice guideline for amyloid PET imaging of the brain 1.0. J. Nucl. Med..

[37] Teune L.K., Bartels A.L., de Jong B.M., Willemsen A.T.M., Eshuis S.A., de Vries J.J., van Oostrom J.C.H., Leenders K.L. (2010). Typical cerebral metabolic patterns in neurodegenerative brain diseases. Mov. Disord..

[38] Brown R.K.J., Bohnen N.I., Wong K.K., Minoshima S., Frey K.A. (2014). Brain PET in suspected dementia: Patterns of altered FDG metabolism. Radiographics.

[39] Yoo S.-W., Ha S., Yoon H., Yoo J.-Y., Lee K.-S., Kim J.-S. (2021). Paradoxical Cerebral Perfusion in Parkinson’s Disease Patients with Orthostatic Hypotension: A Dual-Phase 18F-Florbetaben Positron Emission Tomography Study. J. Parkinsons. Dis..

[40] Friston K.J., Ashburner J., Kiebel S., Nichols T., Penny W.D. (2006). Statistical Parametric Mapping: The Analysis of Funtional Brain Images.

[41] Presotto L., Iaccarino L., Sala A., Vanoli E.G., Muscio C., Nigri A., Bruzzone M.G., Tagliavini F., Gianolli L., Perani D. (2018). Low-dose CT for the spatial normalization of PET images: A validation procedure for amyloid-PET semi-quantification. NeuroImage Clin..

[42] Tzourio-Mazoyer N., Landeau B., Papathanassiou D., Crivello F., Etard O., Delcroix N., Mazoyer B., Joliot M. (2002). Automated anatomical labeling of activations in SPM using a macroscopic anatomical parcellation of the MNI MRI single-subject brain. Neuroimage.

[43] Rolls E.T., Joliot M., Tzourio-Mazoyer N. (2015). Implementation of a new parcellation of the orbitofrontal cortex in the automated anatomical labeling atlas. Neuroimage.

[44] Hort J., O’Brien J.T., Gainotti G., Pirttila T., Popescu B.O., Rektorova I., Sorbi S., Scheltens P. (2010). EFNS guidelines for the diagnosis and management of Alzheimer’s disease. Eur. J. Neurol..

[45] Sala A., Caprioglio C., Santangelo R., Vanoli E.G., Iannaccone S., Magnani G., Perani D. (2020). Brain metabolic signatures across the Alzheimer’s disease spectrum. Eur. J. Nucl. Med. Mol. Imaging.

[46] Soonawala D., Amin T., Ebmeier K.P., Steele J.D., Dougall N.J., Best J., Migneco O., Nobili F., Scheidhauer K. (2002). Statistical parametric mapping of 99mTc-HMPAO-SPECT images for the diagnosis of Alzheimer’s disease: Normalizing to cerebellar tracer uptake. Neuroimage.

[47] McKhann G.M., Knopman D.S., Chertkow H., Hyman B.T., Jack C.R., Kawas C.H., Klunk W.E., Koroshetz W.J., Manly J.J., Mayeux R. (2011). The diagnosis of dementia due to Alzheimer’s disease: Recommendations from the National Institute on Aging-Alzheimer’s Association workgroups on diagnostic guidelines for Alzheimer’s disease. Alzheimer’s Dement..

[48] Pagani M., De Carli F., Morbelli S., Öberg J., Chincarini A., Frisoni G.B., Galluzzi S., Perneczky R., Drzezga A., Van Berckel B.N.M. (2015). Volume of interest-based [18F]fluorodeoxyglucose PET discriminates MCI converting to Alzheimer’s disease from healthy controls. A European Alzheimer’s Disease Consortium (EADC) study. NeuroImage Clin..

