# Supplementary material for: High Correlation of Static First-Minute-Frame (FMF) PET Imaging after 18F-Labeled Amyloid Tracer Injection with [18F]FDG PET Imaging
Source: Sensors (Basel). 2021 Jul 30;21(15):5182. doi: 10.3390/s21155182 (PMC8348394; doi:10.3390/s21155182)
Supplement: Supplementary file 1 [file sensors-21-05182-s001.zip › Sensors-FMF-SM.pdf]

Correlation of static PET data of the first-minute-frame (FMF) after ^{18}F-labelled amyloid tracer injection to [^{18}F]FDG PET images - Supplementary Materials

Alexander P. Seiffert *, Adolfo Gómez-Grande *, Alberto Villarejo-Galende,
Marta González-Sánchez, Héctor Bueno, Enrique J. Gómez and Patricia Sánchez-González

* Co-first author, these authors contributed equally to this work.

S1. Supplementary material to section 2. Materials and methods

S2.1 Supplementary material to section 2.3. Image analysis

Table S1. AAL2 brain regions that compose the grouped VOIs.

Grouped VOI	AAL brain region
Frontal	Precentral gyrus<br>Superior frontal gyrus, dorsolateral<br>Superior frontal gyrus, orbital part<br>Middle frontal gyrus<br>Middle frontal gyrus, orbital part<br>Inferior frontal gyrus, opercular part<br>Inferior frontal gyrus, triangular part<br>Inferior frontal gyrus, orbital part<br>Superior frontal gyrus, medial orbital<br>Superior frontal gyrus, medial<br>Gyrus rectus
Parietal	Postcentral gyrus<br>Superior parietal gyrus<br>Inferior parietal gyrus, excluding supramarginal and angular gyri<br>Supramarginal gyrus<br>Angular gyrus
Occipital	Calcarine fissure and surrounding cortex<br>Cuneus<br>Lingual gyrus<br>Superior occipital gyrus<br>Middle occipital gyrus<br>Inferior occipital gyrus
Temporal	Hippocampus<br>Parahippocampal gyrus<br>Amygdala<br>Fusiform gyrus<br>Heschl's gyrus<br>Superior temporal gyrus<br>Middle temporal gyrus<br>Temporal pole: middle temporal gyrus<br>Inferior temporal gyrus
Anterior cingulate cortex	Anterior cingulate cortex
Posterior cingulate cortex	Posterior cingulate cortex
Precuneus	Precuneus
Striatum	Caudate nucleus<br>Lenticular nucleus, Putamen

S2. Supplementary material to section 3. Results

S2.1 Supplementary material to section 3.2. SUVR analysis

Table S2. Regional SUVRs of [18F]FDG PET and FMFs of Aβ+ patients.

Brain region		SUVR (mean ± SD)							
		Study cohort		[18F]FBP subcohort		[18F]FMM subcohort		[18F]FBB subcohort	
		[18F]FDG	FMF	[18F]FDG	FMF	[18F]FDG	FMF	[18F]FDG	FMF
Frontal	L	0.98 ± 0.15	0.86 ± 0.10	0.95 ± 0.12	0.84 ± 0.09	1.01 ± 0.20	0.90 ± 0.10	0.94 ± 0.13	0.82 ± 0.09
	R	0.99 ± 0.11	0.88 ± 0.07	0.98 ± 0.11	0.87 ± 0.07	1.01 ± 0.11	0.90 ± 0.07	0.93 ± 0.08	0.83 ± 0.05
Occipital	L	1.20 ± 0.16	1.05 ± 0.11	1.17 ± 0.15	1.01 ± 0.11	1.20 ± 0.15	1.08 ± 0.08	1.29 ± 0.23	1.08 ± 0.15
	R	1.19 ± 0.12	1.02 ± 0.11	1.16 ± 0.14	0.97 ± 0.13	1.21 ± 0.07	1.06 ± 0.08	1.30 ± 0.10	1.09 ± 0.04
Parietal	L	0.96 ± 0.15	0.84 ± 0.08	0.92 ± 0.15	0.81 ± 0.08	1.00 ± 0.15	0.88 ± 0.06	1.00 ± 0.19	0.86 ± 0.15
	R	0.89 ± 0.10	0.78 ± 0.07	0.86 ± 0.11	0.76 ± 0.08	0.91 ± 0.08	0.80 ± 0.06	0.91 ± 0.13	0.79 ± 0.09
Temporal	L	0.87 ± 0.16	0.80 ± 0.11	0.81 ± 0.14	0.74 ± 0.10	0.92 ± 0.17	0.85 ± 0.08	0.84 ± 0.15	0.84 ± 0.15
	R	0.88 ± 0.14	0.81 ± 0.10	0.83 ± 0.15	0.76 ± 0.12	0.92 ± 0.12	0.86 ± 0.07	0.93 ±0.07	0.84 ± 0.23
ACC	L	1.02 ± 0.15	0.94 ± 0.11	1.01 ± 0.11	0.93 ± 0.10	1.05 ± 0.18	0.98 ± 0.12	1.00 ± 0.12	0.89 ± 0.10
	R	1.03 ± 0.12	0.93 ± 0.08	1.02 ± 0.09	0.93 ± 0.07	1.04 ± 0.17	0.95 ± 0.10	1.02 ± 0.09	0.90 ± 0.09
PCC	L	1.04 ± 0.20	0.93 ± 0.16	1.03 ± 0.17	0.91 ± 0.15	1.04 ± 0.22	0.96 ± 0.16	1.09 ±0.27	0.90 ± 0.27
	R	0.94 ± 0.18	0.81 ± 0.15	0.94 ± 0.22	0.78 ± 0.18	0.92 ± 0.15	0.84 ± 0.11	1.00 ± 0.15	0.82 ± 0.08
Precuneus	L	1.03 ± 0.18	0.94 ± 0.12	0.98 ± 0.17	0.87 ± 0.08	1.07 ± 0.18	1.03 ± 0.11	1.09 ± 0.20	0.93 ± 0.15
	R	1.05 ± 0.15	0.95 ± 0.13	1.01 ± 0.18	0.89 ± 0.12	1.09 ± 0.11	1.04 ± 0.12	1.09 ± 0.14	0.93 ± 0.11
Striatum	L	0.70 ± 0.18	0.57 ± 0.16	0.71 ± 0.14	0.59 ± 0.13	0.69 ± 0.22	0.56 ± 0.18	0.69 ± 0.26	0.82 ± 0.20
	R	0.81 ± 0.20	0.67 ± 0.18	0.82 ± 0.15	0.68 ± 0.16	0.79 ± 0.24	0.66 ± 0.21	0.86 ± 0.27	0.68 ± 0.20

Table S3. Regional SUVRs of [18F]FDG PET and FMFs of Aβ- patients.

Brain region		SUVR (mean ± SD)							
		Study cohort		[18F]FBP subcohort		[18F]FMM subcohort		[18F]FBB subcohort	
		[18F]FDG	FMF	[18F]FDG	FMF	[18F]FDG	FMF	[18F]FDG	FMF
Frontal	L	1.06 ± 0.16	0.89 ± 0.12	1.10 ± 0.13	0.90 ± 0.08	1.03 ± 0.18	0.86 ± 0.15	1.07 ± 0.12	0.95 ± 0.11
	R	1.09 ± 0.14	0.91 ± 0.09	1.12 ± 0.11	0.92 ± 0.08	1.07 ± 0.16	0.90 ± 0.10	1.07 ± 0.12	0.96 ± 0.12
Occipital	L	1.31 ± 0.12	1.12 ± 0.09	1.33 ± 0.09	1.12 ± 0.08	1.31 ± 0.15	1.12 ± 0.10	1.24 ± 0.06	1.15 ± 0.08
	R	1.28 ± 0.14	1.08 ± 0.10	1.28 ± 0.11	1.06 ± 0.10	1.30 ± 0.16	1.09 ± 0.10	1.20 ± 0.05	1.11 ± 0.09
Parietal	L	1.12 ± 0.10	0.94 ± 0.07	1.12 ± 0.09	0.94 ± 0.07	1.13 ± 0.11	0.95 ± 0.08	1.04 ± 0.07	0.96 ± 0.05
	R	1.05 ± 0.11	0.88 ± 0.08	1.05 ± 0.11	0.87 ± 0.10	1.05 ± 0.12	0.89 ± 0.07	0.97 ± 0.54	0.88 ± 0.05
Temporal	L	1.03 ± 0.12	0.89 ± 0.11	1.04 ± 0.09	0.89 ± 0.08	1.01 ± 0.14	0.88 ± 0.14	1.06 ± 0.09	0.96 ± 0.09
	R	1.04 ± 0.13	0.89 ± 0.11	1.02 ± 0.15	0.86 ± 0.12	1.05 ± 0.12	0.91 ± 0.10	1.04 ± 0.11	0.94 ± 0.12
ACC	L	1.04 ± 0.20	0.90 ± 0.18	1.09 ± 0.13	0.94 ± 0.11	0.98 ± 0.24	0.85 ± 0.22	1.04 ± 0.12	0.98 ± 0.18
	R	1.03 ± 0.17	0.87 ± 0.15	1.08 ± 0.10	0.92 ± 0.08	0.99 ± 0.20	0.73 ± 0.18	1.01 ± 0.11	0.90 ± 0.14
PCC	L	1.28 ± 0.17	1.04 ± 0.10	1.32 ± 0.18	1.03 ± 0.09	1.26 ± 0.16	1.04 ±0.12	1.24 ± 0.24	1.04 ± 0.12
	R	1.13 ± 0.17	0.92 ± 0.13	1.15 ± 0.16	0.89 ± 0.12	1.13 ± 0.18	0.94 ± 0.14	1.03 ± 0.22	0.88 ± 0.13
Precuneus	L	1.24 ± 0.12	1.06 ± 0.08	1.28 ± 0.11	1.05 ± 0.07	1.23 ± 0.13	1.07 ± 0.09	1.16 ± 0.05	1.07 ± 0.03
	R	1.28 ± 0.14	1.08 ± 0.10	1.30 ± 0.15	1.07 ± 0.10	1.28 ± 0.14	1.10 ± 0.10	1.16 ± 0.07	1.08 ± 0.06
Striatum	L	0.80 ± 0.23	0.62 ± 0.20	0.84 ± 0.20	0.66 ± 0.14	0.76 ± 0.26	0.59 ± 0.25	0.82 ± 0.11	0.64 ± 0.08
	R	0.88 ± 0.20	0.70 ± 0.17	0.90 ± 0.19	0.72 ± 0.16	0.86 ± 0.22	0.68 ± 0.19	0.87 ± 0.10	0.76 ± 0.09

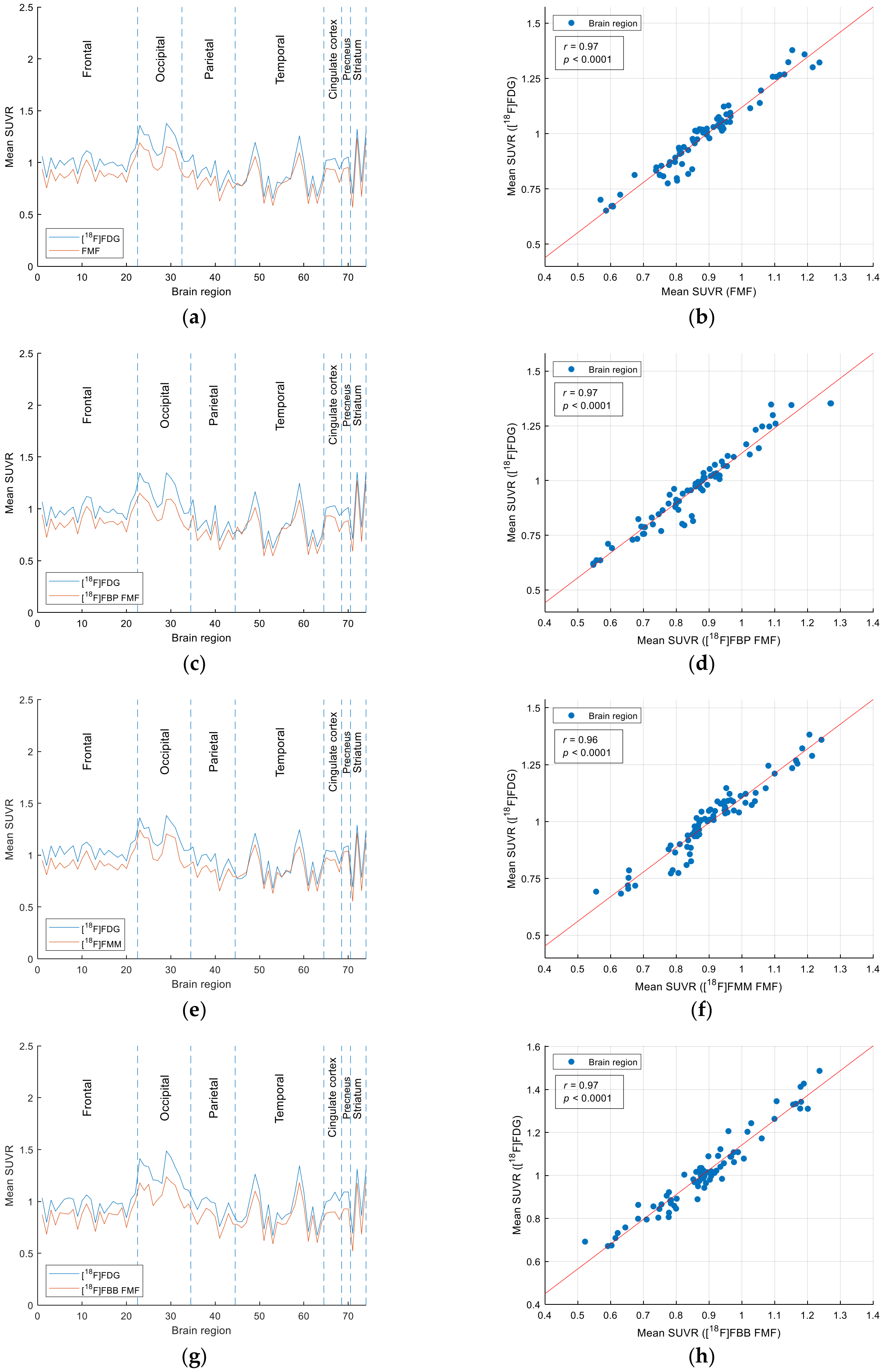

Figure S1. Intensity profiles and correlation charts with least squares line of the mean SUVRs of FMFs and [18F]FDG PET of Aβ+ patients. (**a**), (**b**) Study cohort; (**c**), (**d**) [18F]FBP FMFs; (**e**), (**f**) [18F]FMM FMFs; (**g**), (**h**) [18F]FBB FMFs. Brain regions are sorted as specified in Table S1 (left hemisphere followed by right hemisphere).

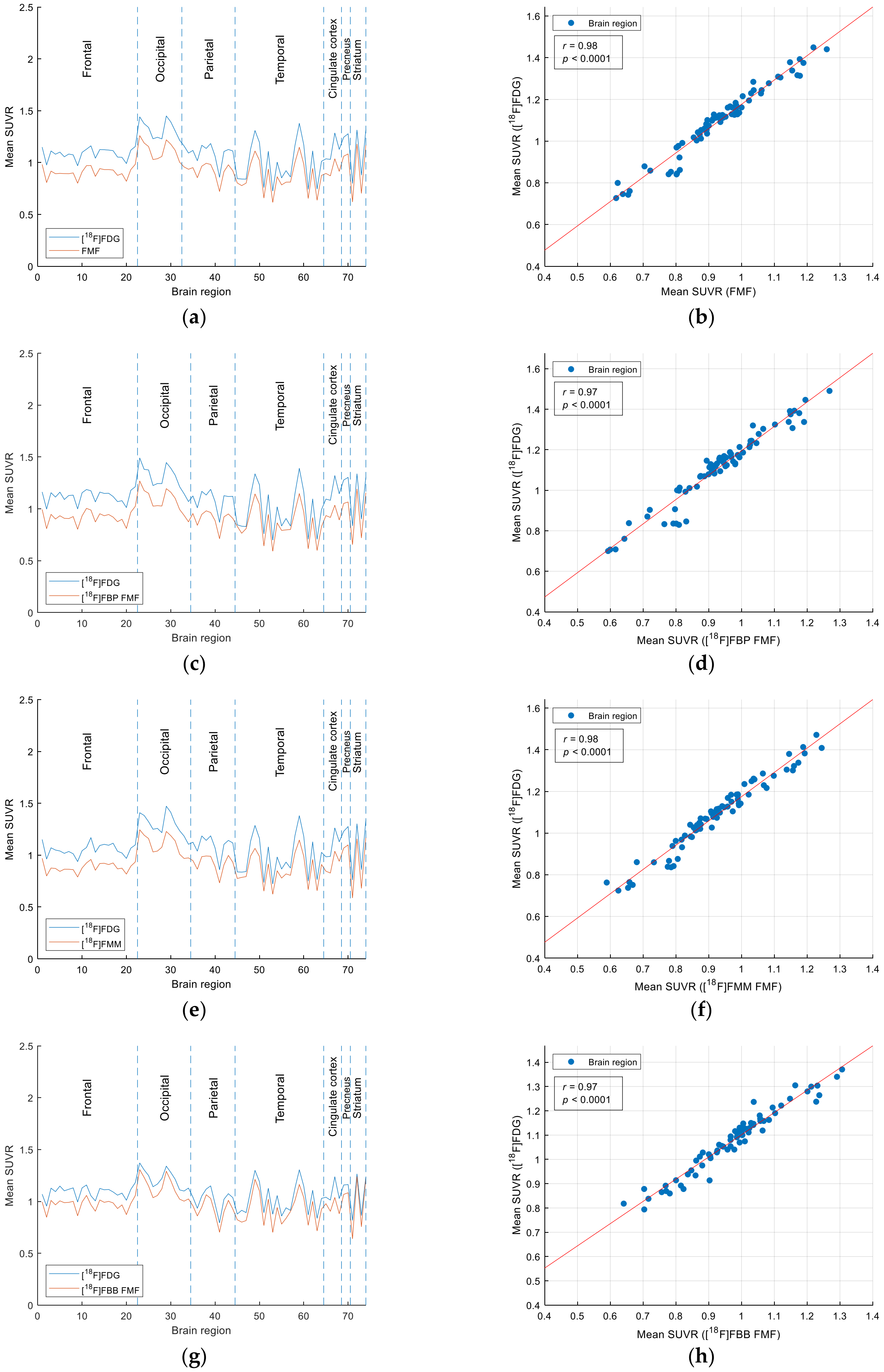

Figure S2. Intensity profiles and correlation charts with least squares line of the mean SUVRs of FMFs and [18F]FDG PET of Aβ- patients. (**a**), (**b**) Study cohort; (**c**), (**d**) [18F]FBP FMFs; (**e**), (**f**) [18F]FMM FMFs; (**g**), (**h**) [18F]FBB FMFs. Brain regions are sorted as specified in Table S1 (left hemisphere followed by right hemisphere).
